# Bond-bending isomerism of Au_2_I_3_^–^: competition between covalent bonding and aurophilicity†
†Electronic supplementary information (ESI) available: Supplementary figures and tables. See DOI: 10.1039/c5sc03568f


**DOI:** 10.1039/c5sc03568f

**Published:** 2015-10-13

**Authors:** Wan-Lu Li, Hong-Tao Liu, Tian Jian, Gary V. Lopez, Zachary A. Piazza, Dao-Ling Huang, Teng-Teng Chen, Jing Su, Ping Yang, Xin Chen, Lai-Sheng Wang, Jun Li

**Affiliations:** a Department of Chemistry & Key Laboratory of Organic Optoelectronics and Molecular Engineering of Ministry of Education , Tsinghua University , Beijing 100084 , China . Email: junli@tsinghua.edu.cn; b Shanghai Institute of Applied Physics , Chinese Academy of Sciences , Shanghai 201800 , China; c Department of Chemistry , Brown University , Providence , Rhode Island 02912 , USA . Email: Lai-Sheng_Wang@brown.edu; d Theoretical Division T-1 , Los Alamos National Laboratory , Los Alamos , NM 87544 , USA

## Abstract

We report a joint photoelectron spectroscopy and theoretical investigation of the gaseous Au_2_I_3_^–^ cluster, which is found to exhibit two types of isomers due to competition between Au–I covalent bonding and Au–Au aurophilic interactions. The covalent bonding favors a bent IAuIAuI^–^ structure with an obtuse Au–I–Au angle (100.7°), while aurophilic interactions pull the two Au atoms much closer, leading to an acutely bent structure (72.0°) with an Au–Au distance of 3.08 Å. The two isomers are separated by a small barrier and are nearly degenerate with the obtuse isomer being slightly more stable. At low temperature, only the obtuse isomer is observed; distinct experimental evidence is observed for the co-existence of a combination of isomers with both acute and obtuse bending angles at room temperature. The two bond-bending isomers of Au_2_I_3_^–^ reveal a unique example of one molecule being able to oscillate between different structures as a result of two competing chemical forces.

## Abstract 2

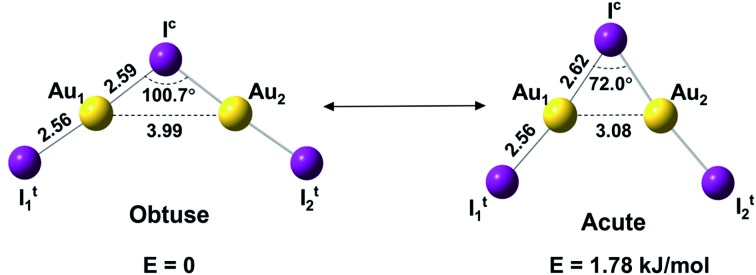
Two isomers, different only by bond angles, are discovered for Au_2_I_3_^–^, due to competition between aurophilic interactions and covalent bonding.

## Introduction

The chemical and physical properties of chemical compounds are dictated by their molecular structures.^1^,^2^ A molecule usually possesses one stable structure at a given condition, because the unique geometric shape of a molecule is determined by its inherent chemical bonding. However, the concept of so-called bond-stretch isomers (BSI) was proposed some time ago, referring to two stable structures with different bond lengths on the same potential energy surface with the same spin-state.^3^–^5^ A number of experimental and theoretical studies were carried out to envisage and characterize the controversial cases of BSI.^6^–^8^ However, most of the claimed BSIs were in solid state or spin-state isomers formed with crossover of different energy surfaces.^9^–^17^ The BSI concept itself still rekindles, even though the early examples of BSI were proven as experimental artifacts.^18^,^19^

In addition to the strong chemical bonding that determines molecular structures, weaker aurophilic interactions have been found in gold systems due to attractions between closed-shell Au^I^ centers.^20^–^31^ Aurophilic interactions, which are intermediate between van der Waals forces and covalent bonding, dominate the structural chemistry of Au^I^ compounds. Strong relativistic effects and dispersion-type electron correlations were found to be the major driving forces of aurophilicity.^22^,^28^–^30^,^32^,^33^

**Fig. 1:**
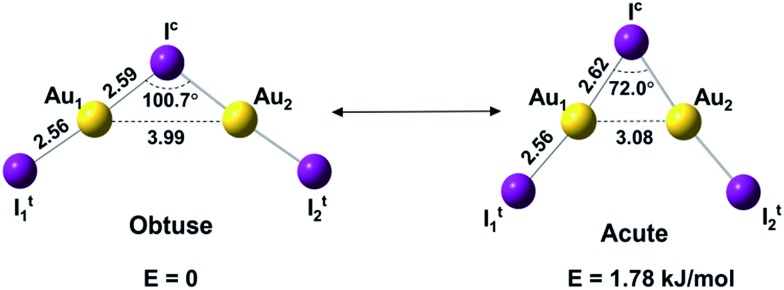
The structures and relative energies of the obtuse and acute isomers of Au_2_I_3_^–^ at the CCSD(T)/AVTZ level. Bond lengths are in Å.

Here we report a joint photoelectron spectroscopy (PES) and quantum chemical study on the gaseous Au_2_I_3_^–^ cluster, in which aurophilic interactions turn out to play a key role. This anion is found to have a *C*_2v_ bent geometric configuration of I–Au–I–Au–I^–^ and exhibit two near-degenerate isomers that we name as “bond-bending isomers (BBIs)”, similar to BSI, which has not been experimentally established heretofore.^34^,^35^ The two BBIs of Au_2_I_3_^–^ reported here differ only in geometry by the bending angle (Fig. 1), owing to different mechanisms of bonding interactions. A prior study showed that the Na_2_Cl_3_^–^ cluster has a linear ground state, because of primarily ionic interactions.^36^ The bent structures of Au_2_I_3_^–^ reflect both the strong Au–I covalency^37^,^38^ and Au–Au aurophilic interactions. The obtuse isomer, which is slightly lower in energy, features a traditional chemical bonding pattern and can be viewed as two AuI moieties linked by a central iodine anion *via* the two Au atoms, whereas the acute isomer with a significantly shorter Au···Au distance features strong aurophilic interactions.

Schmidbaur and Schier predicted that the [E(AuL)_*n*_] type unit could form polyhedra, due to strong aurophilic interactions,^25^ similar to the acute isomer of Au_2_I_3_^–^. Both isomers of Au_2_I_3_^–^ are in singlet spin-states and can inter-convert easily at elevated temperatures because of the low energy barrier between them.

## Experimental methods

Two different experimental apparatuses were used to obtain the photoelectron spectra of the Au_2_I_3_^–^ cluster, which was produced by two completely different methods. It was first produced by electrospray of a 0.1 mM solution of AuI in acetonitrile, and accumulated in a room-temperature ion trap for 0.1 second before being ejected into the extraction zone of a time-of-flight mass spectrometer. The Au_2_I_3_^–^ anions were selected by a mass gate and decelerated before being intercepted by a probe laser beam, 206 nm from a dye laser and 157 nm from an F_2_ excimer laser. The photoelectrons were analyzed by a 2.5 meter long magnetic-bottle electron flight tube, shortened from the original design.^39^ The resolution of the spectrometer was about 3% (Δ*E*_k_/*E*_k_), *i.e.*, about 30 meV for 1 eV electrons.

In the temperature-dependent experiments, the Au_2_I_3_^–^ clusters were produced by laser vaporization of a cold-pressed AuI target in the presence of pure He or a He carrier gas seeded with 5% Ar. The latter was shown previously to give better supersonic cooling.^40^ Clusters formed in the source were entrained by the carrier gas and underwent a supersonic expansion. Anions were extracted from the cluster beam and analyzed by a time-of-flight mass spectrometer. The Au_2_I_3_^–^ anions were selected by a mass gate and decelerated before being detached by a 193 nm laser beam from an ArF excimer laser. Photoelectrons were analyzed by a magnetic-bottle analyzer with an electron energy resolution of ∼2.5% (Δ*E*_k_/*E*_k_).^41^ Clusters with different resident times inside the nozzle were selected as a means to qualitatively control the cluster temperatures.^42^

## Computational methods

Geometric structure optimizations for Au_2_I_3_^–^ were performed using DFT with various functionals, including the generalized gradient approximation (GGA) with the PBE^43^ and PW91 functionals,^44^ the B3LYP hybrid functional,^45^,^46^ the TPSS meta-GGA functional,^47^,^48^ the TPSSh hybrid meta-GGA functional^47^,^48^ and the long-range corrected functionals^49^ with LC-PBE and LC-PW91 and CAM-B3LYP^50^ in Gaussian 09.^51^ Vibrational frequency calculations were carried out to verify that the structures were minima or transition states at equilibrium points of the potential energy surfaces. Zero-point energies (ZPE) were corrected based on the calculated harmonic vibrational frequencies.

In as much as aurophilic interactions required sophisticated electron correlation treatment, high-level *ab initio* calculations were further performed to obtain accurate geometries and energies. We applied the spin-component scaled second-order perturbation theory (SCS-MP2),^52^ coupled clusters with single, double and perturbative triple excitations [CCSD(T)],^53^,^54^ and complete-active-space self-consistent field (CASSCF)^55^ methods. These *ab initio* electron correlation calculations were done with the MOLPRO 2008 program.^56^ Geometry optimizations for the acute and obtuse Au_2_I_3_^–^ structures were both done at the SCS-MP2 and CCSD(T) level. For the neutral Au_2_I_3_ species, single-point energies of the ground and excited states were determined at the optimized anion structures using the CCSD(T) method, which accurately generated the state-specific scalar relativistic energies of all the states needed for simulating the experimental PES. Single-electron VDEs from the anion ground state to the corresponding ground and excited states of the neutral cluster were obtained using the CASSCF/CCSD(T)/SO approach. In both the Gaussian 09 and MOLPRO 2008 calculations, the Stuttgart energy-consistent relativistic pseudopotentials ECP60MDF and ECP28MDF and the corresponding valence basis sets of polarized triple-zeta level (aug-cc-pVTZ-PP) were applied for Au^57^,^58^ and I,^59^ respectively, which were abbreviated as AVTZ.

For chemical bonding analyses, we used the energy decomposition approach (EDA),^60^ various bond order indices, and electron localization function (ELF)^61^ using the PBE exchange–correlation functional implemented in ADF 2013.01.^62^ Here we applied the Slater basis sets with the quality of triple-zeta with two polarization functions (TZ2P).^63^ Frozen core approximation was used for the inner shells of [1s^2^–4f^14^] for Au and [1s^2^–4d^10^] for I. The scalar relativistic (SR) and spin orbit (SO) effects were taken into account by the zero-order-regular approximation (ZORA).^64^

In order to obtain multi-center localized orbitals, we used the Multiwfn^65^ codes to do adaptive natural density portioning (AdNDP) analyses.^66^ The atomic charges were computed *via* natural population analyses (NPA)^67^ by using NBO 3.1 (ref. 68) as implemented in Gaussian 09. The local adiabatic stretching force constants^69^ were calculated using a code written by the Tsinghua group.

## Results

### Photoelectron spectroscopy

**Fig. 2:**
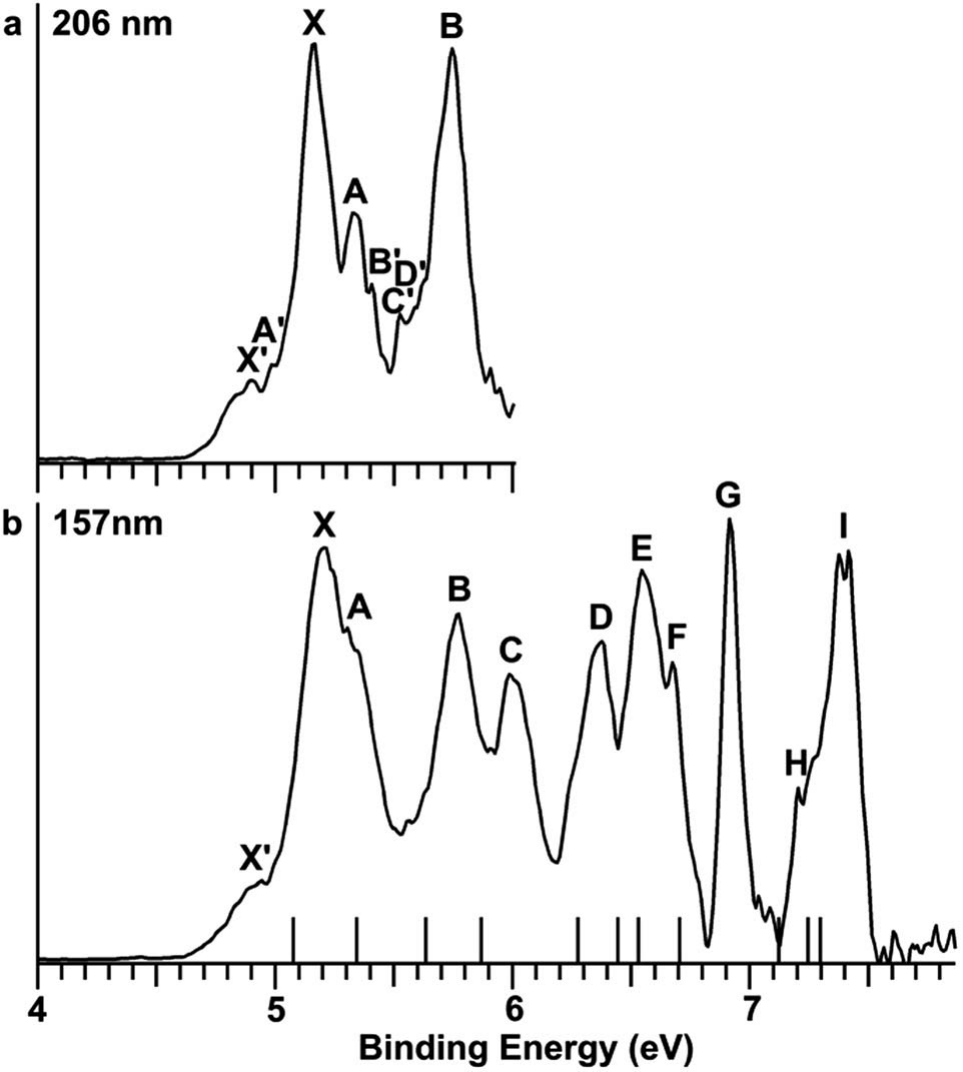
Photoelectron spectra of Au_2_I_3_^–^ at two different photon energies. (a) At 206 nm. (b) At 157 nm. The vertical bars in (b) represent the calculated VDEs from the global minimum obtuse structure at the CASSCF/CCSD(T)/SO level.

The Au_2_I_3_^–^ anion was produced using two different methods and the experiments were carried out using two separate magnetic-bottle PES apparatuses, one equipped with an electrospray ionization (ESI) source^39^ and another with a laser vaporization supersonic cluster source.^41^ With the ESI apparatus, photoelectron spectra were taken under room temperature at two laser wavelengths, 206 nm (6.019 eV) and 157 nm (7.866 eV) (Fig. 2). The room temperature spectra were surprisingly congested. In addition to numerous intense PES features, labeled with the capital letters X and A–I, there were also weak PES features labeled as X′ and A′–D′. The weak intensities of the latter suggested that they might come from another isomer. The binding energies of all the observed features are given in Table S1,† where they are compared with the theoretical results (*vide infra*).

**Fig. 3:**
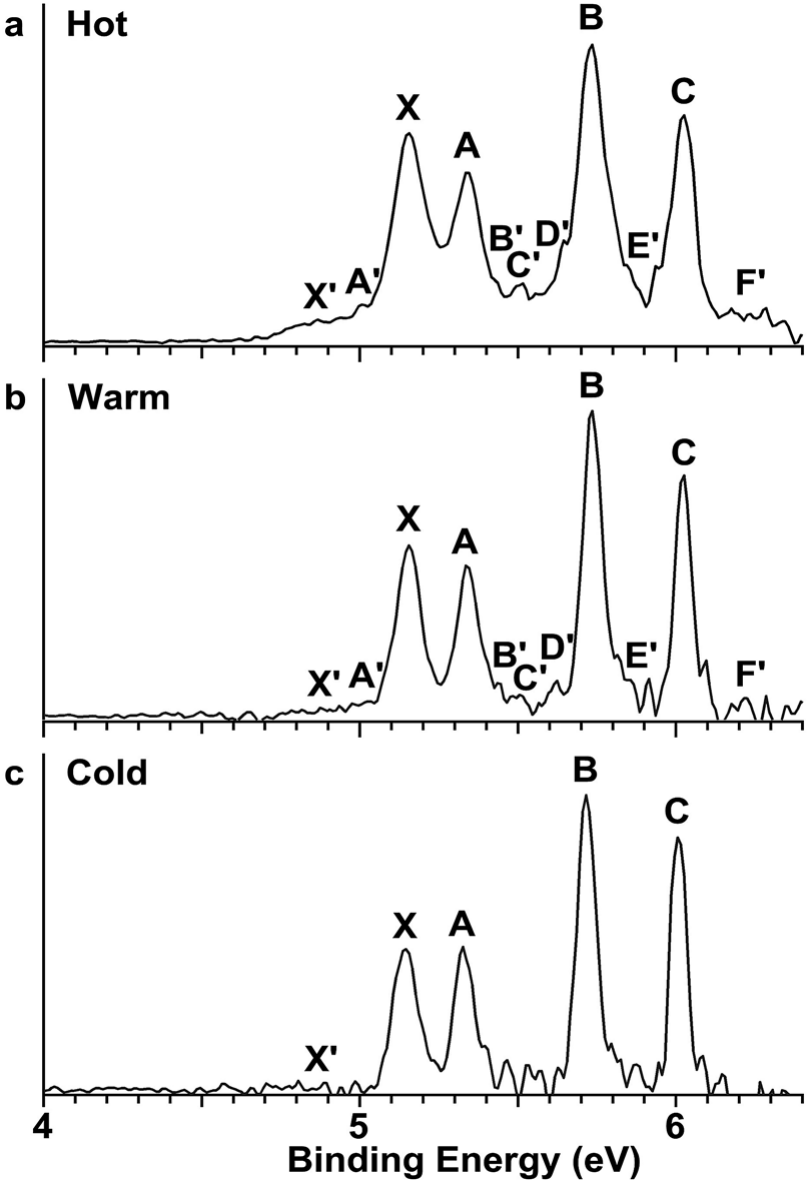
Temperature-dependent photoelectron spectra of Au_2_I_3_^–^ at 193 nm. (a) At short residence time (hot). (b) At medium residence time (warm). (c) At long residence time (cold).

In order to have a better understanding of the congested PES features, temperature-dependent experiments were carried out using a laser vaporization supersonic cluster source.^41^ The Au_2_I_3_^–^ anions were selected and decelerated before being photodetached by the 193 nm (6.424 eV) radiation from an ArF excimer laser at several experimental conditions, as shown in Fig. 3. It was found previously that the residence time of clusters inside the nozzle is an important factor affecting the cluster temperature.^42^ Those clusters that spend more time in the nozzle experience more thermalization collisions with the carrier gas, and they are colder. The residence time of clusters in the nozzle can be controlled to obtain qualitatively hot, warm, and cold clusters.^42^

Photoelectron spectra taken at 193 nm were designated as ‘hot’, ‘warm’, and ‘cold’ in Fig. 3, corresponding to three different residence times. Under the cold condition (Fig. 3c), a well-resolved spectrum with only the four intense features (X, A, B, C) was observed. Under the warm condition (Fig. 3b), weak features (X′, A′–F′) appeared and their intensities increased under the hot condition (Fig. 3a). In addition to the weak PES features observed in Fig. 2 (X′ and A′–D′), two more weak features (E′ and F′) were resolved in Fig. 3. The temperature-dependence of the weak features provided convincing evidence that they were from low-lying isomers, while the intense features were from the most stable isomer.

### Geometries and energetic stabilities of the two BBIs

We used both density functional theory (DFT) and *ab initio* wave function theory (WFT) at different levels (see ESI† for details) to optimize the structures of Au_2_I_3_^–^. The geometries were fully optimized and the results are given in Table S2.† All levels of theory predicted a bent structure, very different from the linear alkali halide system.^36^ Remarkably, we found a very flat potential energy surface with two minima at almost all levels of theory. The geometric parameters at the CCSD(T) level are given in Fig. 1, showing Au_1_···Au_2_ distances of 3.08 Å and 3.99 Å for the acute and obtuse isomers, respectively. All the theoretical results (Table S2†) show that the bond length of Au_1_–I^c^ increases slightly in the acute isomer, while the Au_1_–It1 distance does not change in the two isomers. The geometric parameters obtained by SCS-MP2 agree well with the CCSD(T) calculations, as shown previously.^29^,^70^ However, the numerous DFT methods do not perform well; neither does the popular B3LYP hybrid functional, which even failed to find the acute configuration, nor the long-range corrected CAM-B3LYP functional. Comparably, TPSSh hybrid meta-GGA and PBE performed reasonably well in terms of the covalent bond between Au and I, which was also described in other gold compounds.^71^

**Fig. 4:**
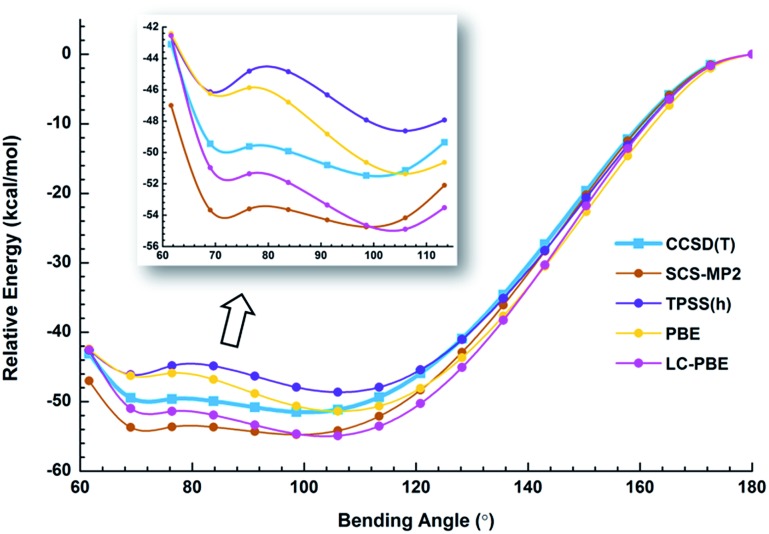
Potential energy curves of Au_2_I_3_^–^ calculated using different methods. All the geometries were obtained by full optimization at each bending angle. Single-point energies for the CCSD(T) and SCS-MP2 methods were carried out using structures optimized at the TPSSh/AVTZ level. The inset shows more details between 60° and 114°.

Because of the inherent aurophilic interactions between the two Au atoms, calculated results from the long-range corrected functionals agree with those from the *ab initio* coupled-cluster method. Thus, high-level quantum chemical methods are crucial in accurately characterizing these nearly degenerate isomers. The full computational results for the energy difference between the acute and obtuse isomers (Δ*E*_A–O_) are given in Table S3.† We found that the linear structure is a transition state, lying about 50 kJ mol^–1^ higher in energy than the two BBIs. Preliminary calculations with the SO-ZORA approach show that SO effects are important for all these species, whereas the relative energies are not affected. Fig. 4 illustrates the rather flat potential energy curve in the region of the two BBIs, which are separated by a small barrier of only 1.78 kJ mol^–1^ at CCSD(T) level. Such a low barrier suggests that the two BBIs can easily inter-convert. Hence, at elevated temperatures many different configurations are expected to co-exist along the bending angle, in excellent agreement with the experimental results, as shown in Table S1† (*vide infra*). The SCS-MP2/AVTZ calculations gave a bending angle of 80.1° for the transition state between the two BBIs. It is interesting to compare the bonding between I_5_^–^ with an apical angle of 94° and the obtuse Au_2_I_3_^–^ isomer,^72^,^73^ because Au can be viewed as a heavy halogen. However, aurophilicity also gives rise to the acute configuration for Au_2_I_3_^–^.

We have estimated the lifetime of the acute BBI at the upper potential well using the Wentzel–Kramers–Brillouin (WKB) escape probability.^74^ Using a bending frequency of 24 cm^–1^, we estimated a lifetime of about 104 ns for the acute BBI, which is sufficiently long-lived to be observed in the photodetachment experiments (Fig. 2 and 3a). A further possibility is that in the condensed-phase the lifetime of the acute isomer could become significantly longer so that separate BBIs could exist therein.

## Discussion

### Comparison of the photoelectron spectra with theoretical results

The photoelectron spectra of Au_2_I_3_^–^ in Fig. 2 and 3 provide definitive experimental evidence for the co-existing isomers at elevated temperatures. Under the cold condition (Fig. 3c), the Au_2_I_3_^–^ cluster resides in its electronic and vibrational ground state with the obtuse structure, giving rise to the intense PES features (X, A–I). Under the hot and warm conditions (Fig. 3a and b), the Au_2_I_3_^–^ cluster can access the acute part of the potential energy surface, giving rise to the weak PES features (X′, A′–F′).

Because the barrier between the two BBIs is low, the broad part of the potential energy curve will be accessed at high vibrational levels or high temperatures, resulting in new spectral features, in remarkable agreement with the experiment. The diffuse X′ low binding energy feature appearing in the hot spectrum by laser vaporization (Fig. 3a) or the room temperature spectrum from ESI (Fig. 2) provides unequivocal evidence for the existence of the acute configurations and for the flatness of the potential energy curve. To understand the spectral features quantitatively, we computed the vertical electron detachment energies (VDEs) of both the acute and obtuse isomers using the CASSCF/CCSD(T)/SO approach, as shown in Table S1.† This approach was shown previously to give reliable theoretical VDEs for Au-containing complexes.^75^–^77^ Theoretical VDEs of the obtuse isomer are plotted as short bars in Fig. 2b for comparison, and they are in excellent agreement with the intense spectral bands (X, A–I). The diffuse and weak low binding energy band X′ corresponds to the acute isomer. Because of the flatness of the potential energy curve, a large range of bond angles is expected to be accessed. Hence, we computed the VDEs for a broad range of bond angles from the obtuse to the acute minima, as given in Table S1.† The calculated VDE of the first detachment channel of the acute isomer increases from 4.828 eV at 72° to 5.015 eV at 90°. This result is in excellent agreement with the experimental VDE of the diffuse band X′, providing the most compelling evidence for the presence of the acute isomer and the shape of the potential energy surface at the CCSD(T) level in Fig. 4. The calculated VDEs for the higher detachment channels are also consistent with the weak features observed at higher temperatures. The excellent agreement between the theoretical VDEs and the temperature-dependent PES provide conclusive evidence for the two BBIs and the validity of the potential energy curve at the CCSD(T) level in Fig. 4.

### Electronic structure and chemical bonding analyses

**Fig. 5:**
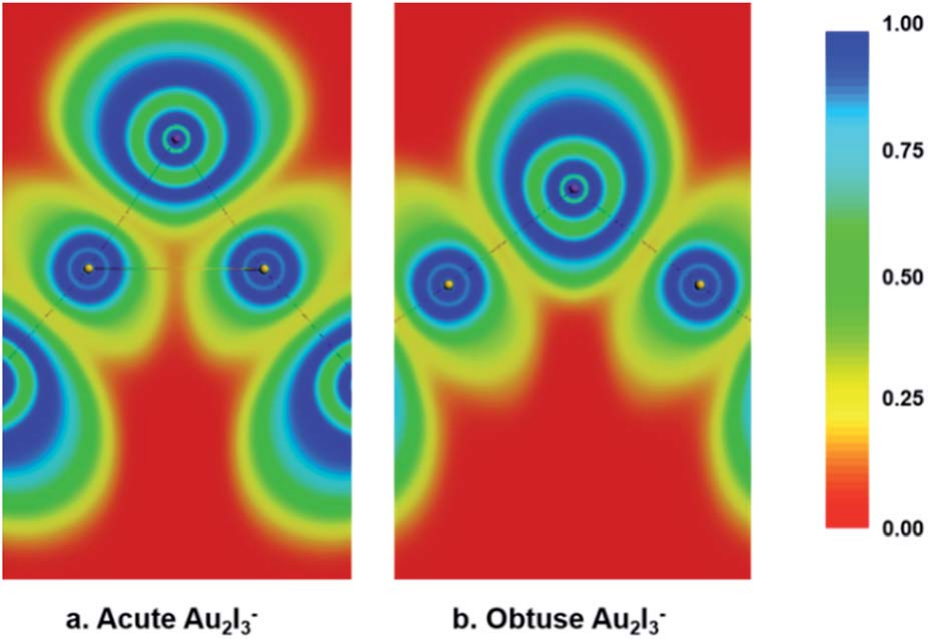
The electron localization functions (ELFs) calculated for both the acute and obtuse Au_2_I_3_^–^ at the PBE/ZORA/TZ2P level. (a) For the acute structure. (b) For the obtuse structure.

We performed various chemical bonding analyses to elucidate the electronic structures of the two BBIs. The chemical bonding can be glimpsed from the electron localization functions (ELFs) shown in Fig. 5. There is discernible Au–Au attraction in the acute isomer in comparison to the obtuse isomer, where no electron-pair density is localized between the two Au sites. Strong covalent bonding can be seen between the central I atom and the neighboring Au atoms in both isomers, but it is slightly stronger in the obtuse isomer. Hence, the Au–Au aurophilic interaction is in competition with the Au–I covalent bonding in the acute isomer. The molecular orbital (MO) contours are depicted for the two isomers in Fig. S2 and S3.† While the upper occupied MOs with non-bonding or anti-bonding features show little change from the obtuse to the acute isomer, the 2b_1_, 3a_1_ and 2a_1_ MOs of the acute isomer represent Au–Au interactions that are not present in the obtuse case. The 1b_1_ MOs in both isomers are dominated by Au_1_(5d)–I^c^(5p)–Au_2_(5d) π-type bonding and have comparable orbital energies, as shown in Fig. S2 and S3.† Especially noteworthy is the different trend of the energy levels of the last two MOs: σ_g_ and σ_u_ for the linear configuration, a_1_ and b_2_ for the bent obtuse or acute structures (Fig. S4†). The 1σ_g_–1a_1_(O)–1a_1_(A) orbital is considerably stabilized in the acute isomer, whereas the 1σ_u_–1b_2_(O)–1b_2_(A) orbital is significantly destabilized in the acute isomer. The different stabilities of the 1a_1_ and 1b_2_ orbitals probably play a key role in the near degeneracy of the two isomers.

As shown in Fig. S4,† the 1a_1_ and 1b_2_ MOs in the acute isomer seem to form two types of three-center two-electron (3c–2e) bonds; one is along the *C*_2_ axis while the other is perpendicular to the *C*_2_ axis, as shown more clearly in the bonding analysis in Fig. S5c′ and d′.† This kind of three-center bonding is also found previously in OC–Th–CO,^78^ similar to IAu–I–AuI^–^ or the Au_2_I_4_^2–^ complex.^79^ These 3c–2e orbitals are conducive to promote Au–Au aurophilic attraction, but weaken the Au–I^c^ interactions, resulting in the more acute angle for Au_1_–I^c^–Au_2_ and elongated Au_1,2_–I^c^ bond length. However, the 1a_1_ and 1b_2_ MOs in the obtuse isomer simply represent two two-center two-electron (2c–2e) Au_1,2_–I^c^ bonds, as shown in Fig. S5c and d.† Therefore, the Au–Au aurophilic interactions in the acute isomer involve several MOs.

The calculated bond orders based on various theoretical schemes and adiabatic stretching force constants in both the acute and obtuse conformers are given in Table S4.† These results also give strong evidence that the Au(i)···Au(i) aurophilic attraction becomes stronger at the cost of weakening the covalent bonding between the central I and the adjacent Au in the acute isomer. All methods give a larger Au–Au bond order and a smaller Au–I^c^ bond order in the acute isomer relative to the obtuse isomer. The Au–Au and Au–I^c^ stretching frequencies show a similar trend. Table 1 provides results of the energy decomposition analysis (EDA) with SO coupling correction, showing the key role of the covalent chemical bonding interaction according to orbitals with different irreducible representations. Steric interactions in the linear, obtuse and acute isomers are almost identical. However, the orbital interactions of the b_2_ MOs in the obtuse isomer are 1.15 eV stronger than that in the acute isomer. On the other hand, the orbital interactions of the a_1_ MOs in the acute isomer are 1.04 eV more stable than the obtuse isomer, suggesting that the energy difference of these two isomers is mainly due to the energy competition between the b_2_ and a_1_ orbitals. It is also shown that the contribution of the SO coupling effect to the total bonding energy is significant (6.40 eV), but the correction is the same for the isomers with different bending angles (Table 1). Accordingly, the relative energetic stability is not influenced by the SO correction.

**Table 1:** Energy decomposition analyses (EDA) for the acute, obtuse and linear Au_2_I_3_^–^ at the PBE/ZORA/TZ2P level of theory^*a*^

	Steric role^*b*^	Orbital interaction^*c*^					Total bonding energy^*d*^	
		a_1_	b_1_	b_2_	a_2_	Sum	SR	SO
Acute	11.16	–10.71	–1.84	–8.99	–3.83	–25.37	–14.21	–20.61
Obtuse	11.29	–9.67	–1.91	–10.14	–3.82	–25.54	–14.25	–20.65
Linear	11.23	–13.52	–5.72	–5.72	–0.01	–24.97	–13.74	–20.14

^*a*^All energies are given in eV. The SO single-point calculations are based on the spin-restricted fragments of the SR results at the equilibrium geometries.

^*b*^The sum of electrostatic and Pauli interactions.

^*c*^Each irreducible representation means the sum of the contributions from that orbital type. There are 11a_1_, 10b_1_, 5b_2_ and 4a_2_ orbitals that are summed up.

^*d*^Sum of the steric and orbital interactions.

## Conclusions

In summary, we have observed two bond-bending isomers for gaseous Au_2_I_3_^–^, which are only different by the IAu–I–AuI bond bending angles as a result of the competition between Au–I covalent bonding and Au–Au aurophilic interactions. High-level theoretical calculations showed that the acute and obtuse isomers are nearly degenerate in energy and separated by a small energy barrier with the obtuse isomer slightly lower in energy. Temperature-dependent photoelectron spectra were obtained for Au_2_I_3_^–^, which was produced with two completely different experimental methods, with clear evidence for the presence of these isomers. Systematic chemical bonding analyses revealed the subtle differences in the bonding interactions of the two types of structural isomers. The current finding presents a rare example of structural isomerism, akin to the long proposed concept of bond-stretching isomers.

## Supplementary Material

Supplementary information
