# Supplementary material for: Bond-bending isomerism of Au2I3–: competition between covalent bonding and aurophilicity
Source: Chem Sci. 2015 Oct 13;7(1):475–81. doi: 10.1039/c5sc03568f (PMC5952886; doi:10.1039/c5sc03568f)
Supplement: Supplementary file 1 [file SC-007-C5SC03568F-s001.pdf]

Electronic Supplementary Material (ESI) for Chemical Science.
This journal is © The Royal Society of Chemistry 2015

Supporting Information for

Bond-Bending Isomerism of $Au_2I_3^-$: Competition between Covalent Bonding and Aurophilicity

Wan-Lu Li,[a] Hong-Tao Liu,[b] Tian Jian,[c] Gary V. Lopez,[c] Zachary A. Piazza,[c] Dao-Ling Huang,[c] Teng-Teng Chen,[c] Jing Su,[a,b] Ping Yang,[d] Xin Chen,[a] Lai-Sheng Wang,*[c] and Jun Li*[a]

[a] Department of Chemistry & Key Laboratory of Organic Optoelectronics and Molecular Engineering of Ministry of Education, Tsinghua University, Beijing 100084, China.

[b] Shanghai Institute of Applied Physics, Chinese Academy of Sciences, Shanghai 201800, China

[c] Department of Chemistry, Brown University, Providence, Rhode Island 02912, USA.

[d] Theoretical Division T-1, Los Alamos National Laboratory, Los Alamos, NM 87544, USA.

*E-mail: Lai-Sheng_Wang@brown.edu; junli@tsinghua.edu.cn

Contents

Table S1. Experimental VDEs of $Au_2I_3^-$ compared with theoretical VDEs S2
Table S2. Structural parameters of the acute (A) and obtuse (O) isomers S3
Table S3. The energy difference between the two isomers and aurophilic interaction energies S3
Table S4. bond orders and local adiabatic stretching force constant S3
Figure S1. Potential energy curves for the isomerization at different levels of theory S4
Figure S2. The occupied molecular orbital contours of the acute isomer S4
Figure S3. The occupied molecular orbital contours of the obtuse isomer S5
Figure S4. Orbital energy-level correlation diagram for the occupied MOs S5
Figure S5. AdNDP bonding analyses for the obtuse and acute structures S6
References S6

Table S1. Experimental VDEs of $Au_2I_3^-$ compared with theoretical VDEs, calculated with the CASSCF/CCSD(T)/SO method for structures with different bending angles. All energies are in eV.

Features[a]	Exp. VDEs[b]	Theo. VDEs[c]						
		100.7°(O) [d]	90°	85°	81°	78°	75°	72°(A)[e]
X′	4.86(5)		5.015	4.977	4.938	4.905	4.868	4.828
A′	5.00(5)		5.184	5.156	5.134	5.117	5.101	5.087
X	5.16(4)	5.073						
A	5.34(3)	5.343						
B′	5.45(5)					5.480	5.454	5.431
C′	5.51(5)		5.574	5.538	5.505			
D′	5.63(5)		5.804	5.772	5.746	5.727	5.710	5.689
B	5.74(3)	5.736						
E′	~5.9			6.187	6.141	6.098	6.048	5.907
C	6.03(3)	5.869						
F′	~6.2		6.228	6.343	6.309	6.279	6.250	6.278
D	6.37(5)	6.277	6.384					
E	6.55(5)	6.443	6.486	6.462	6.446	6.430	6.415	6.532
F	6.67(5)	6.531	6.691	6.693	6.701	6.706	6.713	6.649
G	6.91(5)	6.702	7.094	7.079	7.070	7.058	7.036	7.016
H	7.21(5)	7.124	7.171	7.130	7.097	7.073	7.062	7.023
I	7.39(5)	7.246	7.265	7.257	7.255	7.262	7.273	7.294
		7.298						

[a] X, A, B, C and X′, A′, B′, C′, D′, E′, F′ are measured from the 193 nm spectra; D, E, F, G, H and I are measured from the 157 nm spectrum.

[b] The number in the parentheses indicates the uncertainty in the last digit.

[c] Theoretical VDEs with bending angles ranging from the acute to the obtuse. Geometries are optimized at CCSD(T)/AVTZ level with the fixed bending angles.

[d] Obtuse structure with 100.7° bending angle.

[e] Acute structure with 72.0° bending angle.

Table S2. Structural parameters of the acute (A) and obtuse (O) isomers of $Au_2I_3^-$ at different levels of theory.

	Method	Au_1-Au_2 (Å)		Au_1-I^c (Å)		Au_1-I_1^t (Å)		Au_1-I^c-Au_2 (°)		I^c-Au_2-I_2^t (°)	
		A	O	A	O	A	O	A	O	A	O
Post-HF	CCSD(T)	3.08	3.99	2.62	2.59	2.56	2.56	72.0	100.7	174.9	177.5
	SCS-MP2	3.03	3.92	2.60	2.57	2.54	2.54	71.3	99.2	175.2	177.7
GGA	PBE	2.99	4.17	2.64	2.60	2.58	2.57	69.0	106.3	172.8	176.9
	PW91	2.98	4.16	2.64	2.60	2.58	2.57	68.7	106.2	172.8	176.9
Hybrid-GGA	B3LYP	-	4.22	-	2.63	-	2.59	-	106.6	-	176.9
Meta-GGA	TPSS	2.90	4.18	2.64	2.60	2.57	2.57	66.5	106.8	171.4	176.9
Hybrid-Meta -GGA	TPSSh	2.94	4.15	2.63	2.60	2.57	2.56	67.8	106.2	172.3	177.0
Long-range	CAM-B3LYP	-	4.15	-	2.61	-	2.57	-	105.5	-	177.2
corrected	LC-PBE	3.03	3.98	2.55	2.53	2.51	2.51	73.1	103.8	175.0	177.5
	LC-PW91	3.06	3.99	2.55	2.53	2.51	2.51	73.9	103.9	175.3	177.5

Table S3. The energy difference between the acute and obtuse isomers of $Au_2I_3^-$ and aurophilic interaction energies calculated at various levels of theory. All energies in kJ/mol are corrected for zero-point energies (ZPE).[a]

	CCSD(T)	SCS-MP2	PBE	PW91	LC-PBE	LC-PW91	TPSS	TPSSh
E_a[b]	0.25	0.29	0.33	0.42	0.08	0.08	2.76	1.55
E_{AI}[c]	49.20	53.63	45.69	45.94	51.00	50.58	47.23	45.69
$\Delta E_{A\text{-}O}$[d]	1.78	1.10	5.02	4.77	3.34	3.51	1.21	2.38

[a] ZPE values of CCSD(T) and SCS-MP2 methods are estimated from TPSSh/AVTZ results.

[b] Activation energies from the acute structure to obtuse structure.

[c] The aurophilic interaction (AI) energy calculated by the energy of the linear $Au_2I_3^-$ minus the acute one.

[d] The energy difference between the acute and obtuse isomers.

Table S4. Calculated bond orders and local adiabatic stretching force constant between Au and I in both stable acute and obtuse conformers.

	Structure	Mayer[1]	G-J[2]	$N\text{-}M_{(1)}$[3, 4]	$N\text{-}M_{(2)}$	$N\text{-}M_{(3)}$	Force const. (mDyn/Å)	Freq. (cm^{-1})
Au_1-I^c	Acute	0.67	0.43	0.50	0.53	0.53	0.900	140.7
	Obtuse	0.74	0.47	0.55	0.57	0.57	1.218	163.7
Au_1-I_1^t	Acute	0.88	0.62	0.85	0.83	1.00	1.480	180.4
	Obtuse	0.88	0.62	0.87	0.84	1.03	1.506	182.0
Au_1-Au_2	Acute	0.22	0.11	0.11	0.09	0.10	0.084	38.1
	Obtuse	0.02	0.03	0.03	0.01	0.02	0.064	33.2

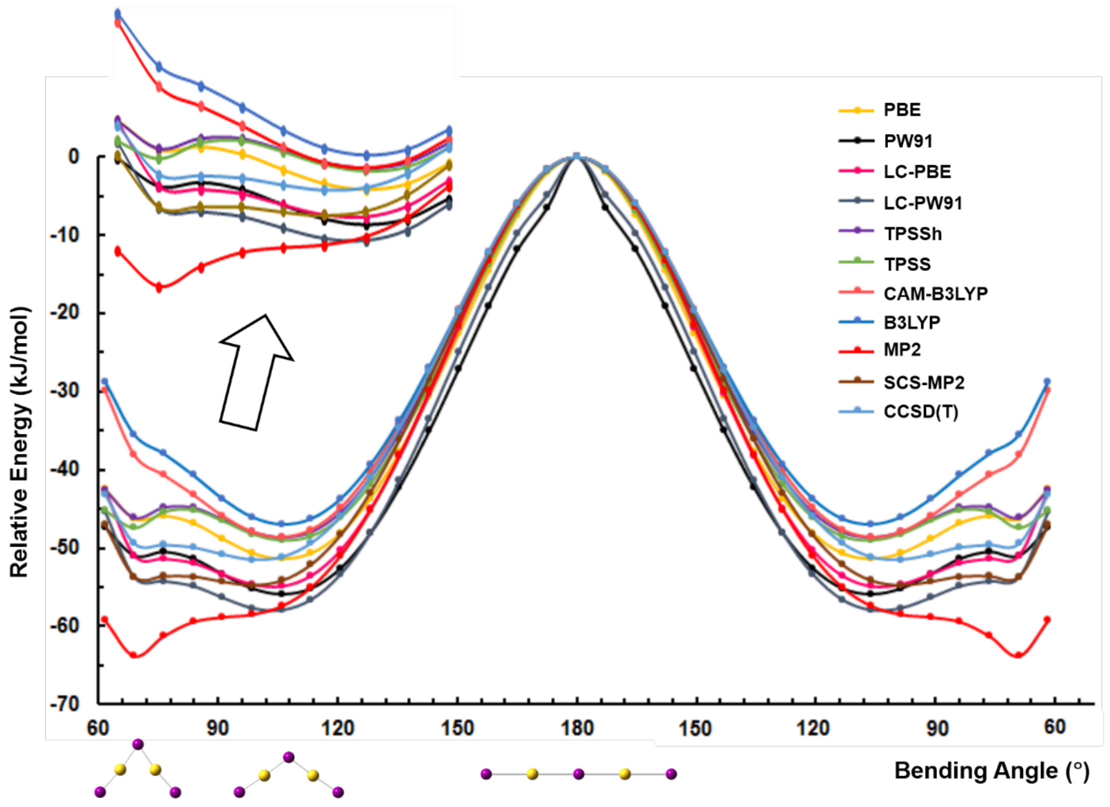

Figure S1. Potential energy curves of $Au_2I_3^-$ as a function of the bond angle at different levels of theory. All the geometries are obtained by full optimization at each bending angle. Single-point energies for the CCSD(T) and SCS-MP2 methods are carried out using the structures optimized at the TPSSh/AVTZ level.

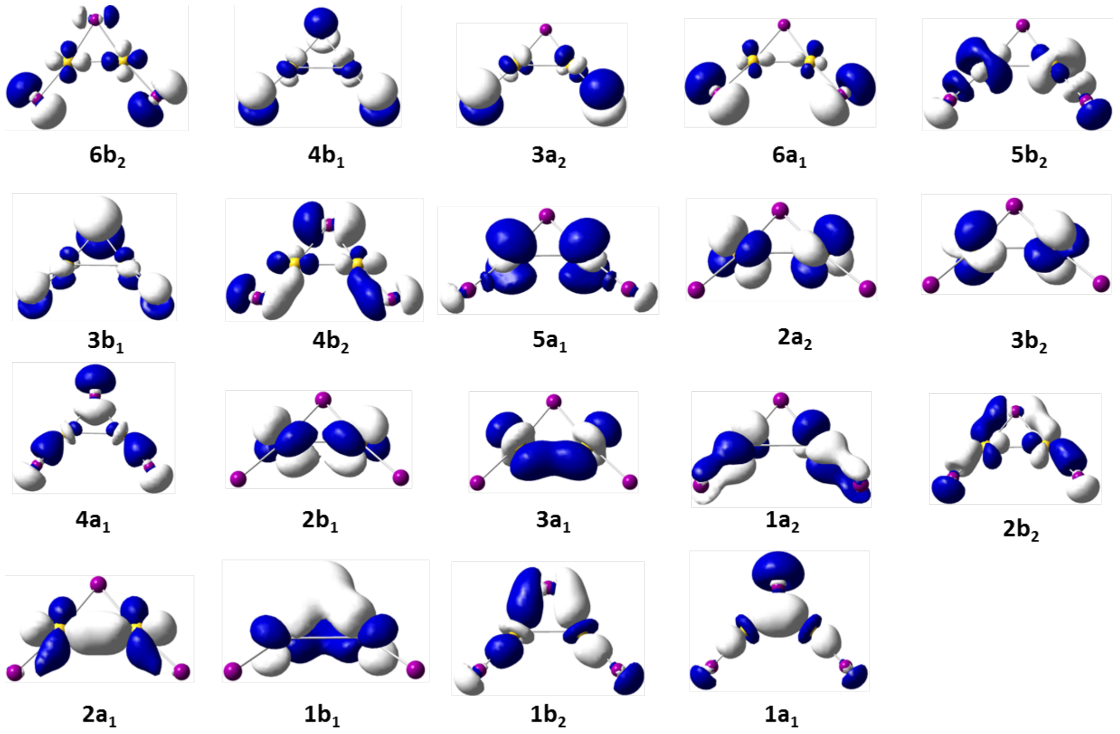

Figure S2. The occupied MO contours of the acute $Au_2I_3^-$ isomer at the PBE/TZ2P level.

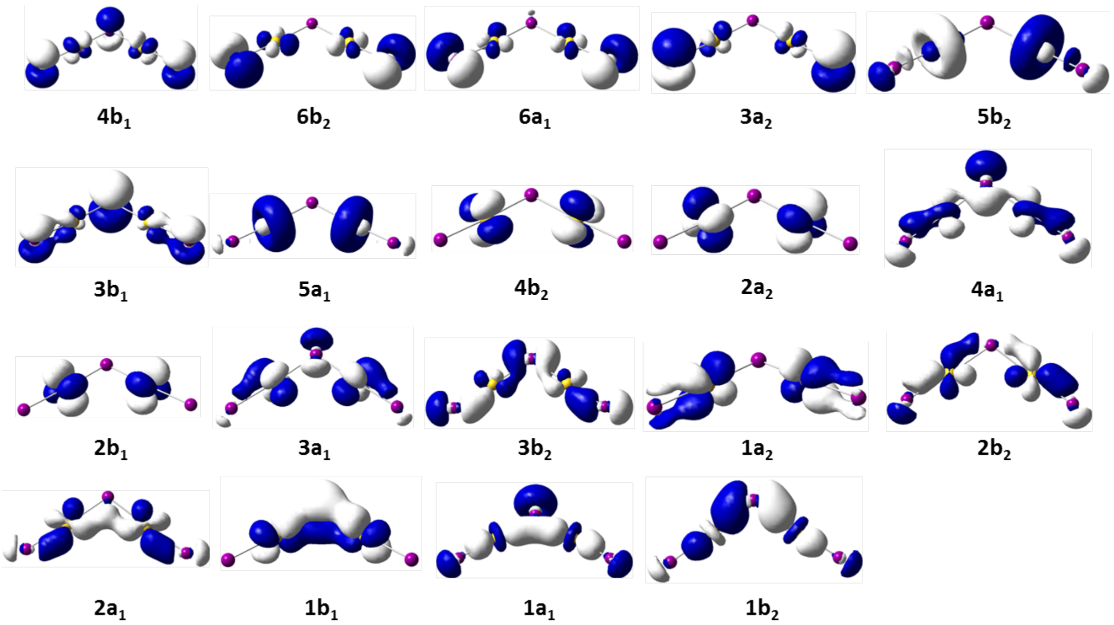

Figure S3. The occupied MO contours of the obtuse $Au_2I_3^-$ isomer at the PBE/TZ2P level.

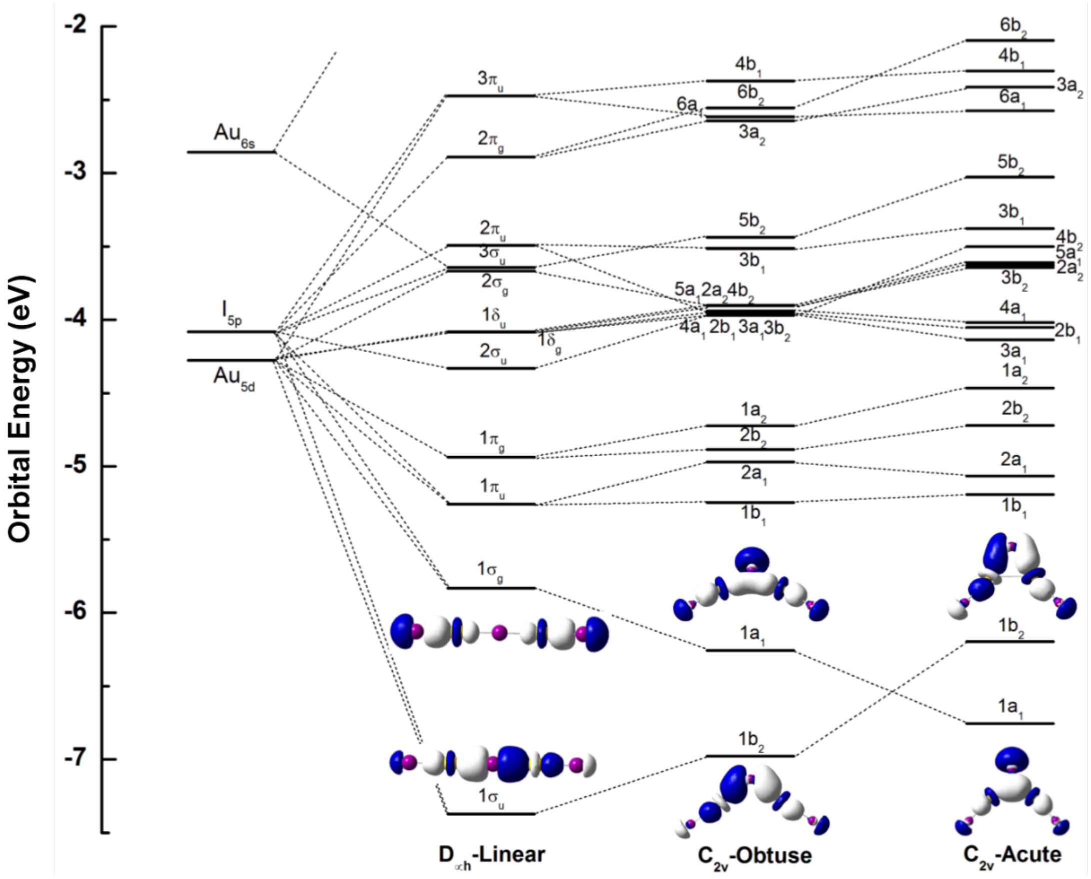

Figure S4. Orbital energy-level correlation diagram for the occupied MOs of the two BBI minima and at the linear transition state. Thee I-5p atomic orbital level is shifted to align with the $1\delta_u$ nonbonding orbital.

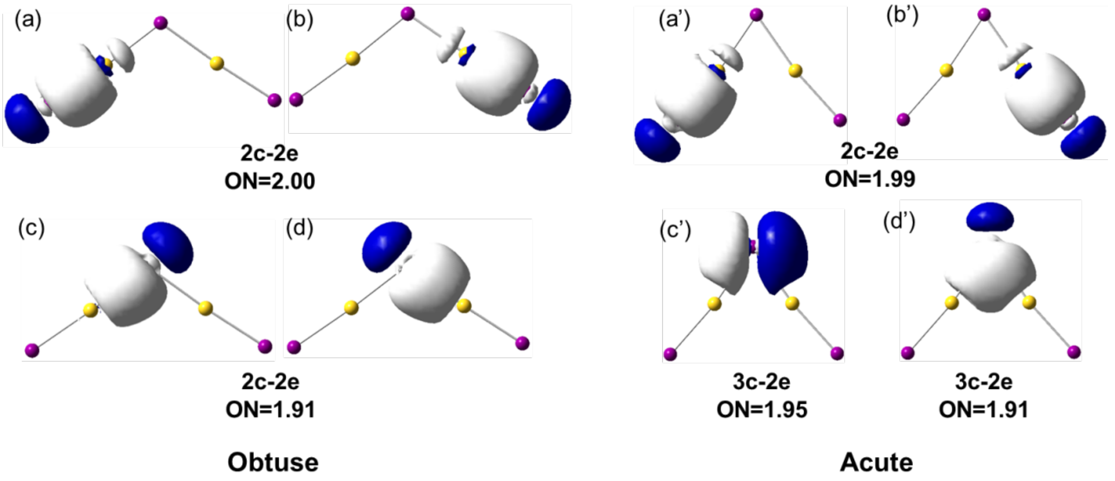

Figure S5. Results of the adaptive natural density partitioning (AdNDP) analyses for the obtuse and acute structures (Isosurface value = 0.04). ON = occupation number.

References:

1 I. Mayer, *Chem. Phys. Lett.*, 1983, **97**, 270-274.

2 M. Gopinathan and K. Jug, *Theor. Chim. Acta*, 1983, **63**, 497-509.

3 A. Michalak, R. L. DeKock and T. Ziegler, *J. Phys. Chem. A*, 2008, **112**, 7256-7263.

4 R. F. Nalewajski, J. Mrozek and A. Michalak, *Int. J. Quantum Chem.*, 1997, **61**, 589-601.
